# Predicting COVID-19—Comorbidity Pathway Crosstalk-Based Targets and Drugs: Towards Personalized COVID-19 Management

**DOI:** 10.3390/biomedicines9050556

**Published:** 2021-05-17

**Authors:** Debmalya Barh, Alaa A. Aljabali, Murtaza M. Tambuwala, Sandeep Tiwari, Ángel Serrano-Aroca, Khalid J. Alzahrani, Bruno Silva Andrade, Vasco Azevedo, Nirmal Kumar Ganguly, Kenneth Lundstrom

**Affiliations:** 1 Centre for Genomics and Applied Gene Technology, Institute of Integrative Omics and Applied Biotechnology (IIOAB), Nonakuri, Purba Medinipur 721172, India; 2 Departamento de Genética, Ecologia e Evolução, Instituto de Ciências Biológicas, Universidade Federal de Minas Gerais, Belo Horizonte 31270-901, Brazil; sandip_sbtbi@yahoo.com (S.T.); vascoariston@gmail.com (V.A.); 3 Department of Pharmaceutics and Pharmaceutical Technology, Faculty of Pharmacy, Yarmouk University, Irbid 21163, Jordan; alaaj@yu.edu.jo; 4 School of Pharmacy and Pharmaceutical Science, Ulster University, Coleraine BT52 1SA, UK; m.tambuwala@ulster.ac.uk; 5 Biomaterials and Bioengineering Lab, Centro de Investigación Traslacional San Alberto Magno, Universidad Católica de Valencia San Vicente Mártir, 46001 Valencia, Spain; angel.serrano@ucv.es; 6 Department of Clinical Laboratories Sciences, College of Applied Medical Sciences, Taif University, Taif 21944, Saudi Arabia; ak.jamaan@tu.edu.sa; 7 Laboratório de Bioinformática e Química Computacional, Departamento de Ciências Biológicas, Universidade Estadual do Sudoeste da Bahia (UESB), Jequié 45206-190, Brazil; bandrade@uesb.edu.br; 8 National Institute of Immunology, Aruna Asaf Ali Marg, Jawaharlal Nehru University, New Delhi 110067, India; nkganguly@nii.ac.in; 9 Institute of Liver and Biliary Science, New Delhi 110070, India; 10 Policy Center for Biomedical Research, Translational Health Science & Technology Institute, Faridabad 121001, India; 11 PanTherapeutics, CH 1095 Lutry, Switzerland

**Keywords:** COVID-19, comorbidity, shared pathways, drug targets, personalized therapy

## Abstract

It is well established that pre-existing comorbid conditions such as hypertension, diabetes, obesity, cardiovascular diseases (CVDs), chronic kidney diseases (CKDs), cancers, and chronic obstructive pulmonary disease (COPD) are associated with increased severity and fatality of COVID-19. The increased death from COVID-19 is due to the unavailability of a gold standard therapeutic and, more importantly, the lack of understanding of how the comorbid conditions and COVID-19 interact at the molecular level, so that personalized management strategies can be adopted. Here, using multi-omics data sets and bioinformatics strategy, we identified the pathway crosstalk between COVID-19 and diabetes, hypertension, CVDs, CKDs, and cancers. Further, shared pathways and hub gene-based targets for COVID-19 and its associated specific and combination of comorbid conditions are also predicted towards developing personalized management strategies. The approved drugs for most of these identified targets are also provided towards drug repurposing. Literature supports the involvement of our identified shared pathways in pathogenesis of COVID-19 and development of the specific comorbid condition of interest. Similarly, shared pathways- and hub gene-based targets are also found to have potential implementations in managing COVID-19 patients. However, the identified targets and drugs need further careful evaluation for their repurposing towards personalized treatment of COVID-19 cases having pre-existing specific comorbid conditions we have considered in this analysis. The method applied here may also be helpful in identifying common pathway components and targets in other disease-disease interactions too.

## 1. Introduction

The current outbreak of COVID-19 has raised unprecedented obstacles for all and has broken down the healthcare system across several countries [1,2,3,4,5]. According to the WHO weekly epidemiological update on COVID-19 (4 May 2021), COVID-19 is accounted for 151,812,556 cases and 3,186,817 deaths worldwide [6]. Heterogeneity is the most striking feature of the outbreak, ranging from no symptoms to critical conditions and death [7,8,9]. The risk of serious disease is increased by age, gender, and comorbidities [10,11,12]. The seriousness of COVID-19 disease progression is also associated with several chronic comorbid conditions [13,14]. Worst outcomes have been correlated with comorbidities, which include hypertension, diabetes, cardiovascular diseases (CVDs), obesity, chronic kidney diseases (CKDs), cancers, and chronic obstructive pulmonary disease (COPD), among others [8,15,16,17]. Hypertension, CVDs, and diabetes mellitus are the most common comorbidities in COVID-19 [16,18]. Further, several post- and long-term complications in COVID-19 survivors are also mounting with time [19].

Although several drugs have been tested for repurposing in COVID-19 therapy and several drugs are under clinical trials [20,21,22], none of these drugs have proven to be the gold standard to treat the disease. The treatment becomes more complex while considering the comorbid conditions due to the lack of comorbidity assessment, drug–drug interactions [23], and most importantly, lack of knowledge of the pathway crosstalk between COVID-19 and its comorbid conditions to understand the complexity in pathogenesis. The treatment’s efficacy could be increased if the molecular pathway crosstalk between the specific comorbidity and COVID-19 is understood and that crosstalk-based targets and targeting drugs are used towards personalized medication. Although, some reports are available such as crosstalk between COVID-19 and breast cancer [24,25], COVID-19 and Alzheimer’s disease [26], COVID-19 and ischemic stroke [27], hyperthyroidism and COVID-19 [28], COVID-19 and age, and COVID-19 and hypertension [29]; omics-based molecular pathway crosstalk between COVID-19 and its associated vital comorbid conditions and those crosstalk-based targets have not been investigated.

In this study using multi-omics data sets and bioinformatics approaches, we attempted to understand the pathway crosstalk between specific comorbid conditions and COVID-19 and that crosstalk-based targets towards personalized management of COVID-19 with pre-existing specific comorbidity. We have considered five key comorbid conditions (hypertension, diabetes, CVDs, CKDs, and cancers) in this analysis.

## 2. Materials and Methods

Since there is not much information about the pathway–pathway interactions of COVID-19 with its associated comorbid conditions, we applied a pathway mapping approach to understand the molecular crosstalk between COVID-19 and its comorbid diseases. Recently, we achieved high precision in predicting COVID-associated symptoms, diseases, and drugs [30,31] using five omics data sets: interactome data from Gordon et al. 2020 [32], proteome data from Bojkova et al. 2020 [33], transcriptome profiles from Blanco-Melo et al. 2020 [34] and Xiong et al. 2020 [35], and bibliome data (COVID-19 associated genes from DisGeNET data collection (https://www.disgenet.org/covid/genes/summary/ (accessed on 12 March 2021)). We used these same data sets also in this study. Unlike our previous bioinformatics approaches [30,31], here, we have combined these multi-omics data together (total 2163 genes) and used them in this analysis.

First, we identified the genes associated with the key comorbid disease pathways from KEGG [36] and MSigDB [37] databases to understand the pathway–pathway interactions or crosstalks. From KEGG, diabetes (hsa04930, 47 genes), hypertension (hsa04614, 23 genes), and cancer (hsa05200, 395 genes), and COVID-19 (hsadd05171, 232 genes) pathway genes were collected. Since the CVD and CKD pathways are not available in KEGG, we used the DisGeNET database [38] to collect CVD- (1551 genes) and CKD- (908 genes) associated genes. Next, we used the Vinny tool (https://bioinfogp.cnb.csic.es/tools/venny/ (accessed on 18 March 2021)) to identify the three gene sets’ core common genes: multi-omics, KEGG- COVID-19 (hsadd05171), and the specific comorbid disease pathway genes. The three data sets’ core common genes are then used to identify the pathways in which these genes are involved using the TopFun algorithm of ToppGene suit [39] and Enrichr [40]. In Enrichr, Reactome, KEGG 2019 human pathway, and MSigDB hallmark 2020 databases were used. The most common pathways enriched within the top ten pathways from each database were used for the results. For common pathway-based target identification, the candidate gene prioritization option of ToppGene [39] was used. In this analysis, the common core genes were used as a test set, and the remaining multi-omics data and KEGG COVID-19 pathway genes were used as a training set. Enrichr was also employed for PPI-based hub gene identification using the common core genes, and interactive [41] was used for comorbidity-specific target identification from the comorbidity-specific hub-gene sets. Finally, we used Drug Target Profiler (DTP) [42] to identify approved drugs for all our identified targets.

## 3. Results

### 3.1. Pathway Crosstalks in Diabetes and COVID-19

The KEGG diabetes pathway (hsa04930) was used to understand pathway–pathway interactions between diabetes and COVID-19. Following the methods described, we found that 21 genes (*TNF*, *MAPK1*, *MAPK8*, *PIK3CA*, *PIK3CB*, *PIK3CD*, *MAPK3*, *INSR*, *HK3*, *HK2*, *PKM*, *INS*, *MTOR*, *PIK3CG*, *ADIPOQ*, *PIK3R1*, *PIK3R2*, *PIK3R3*, *MAPK10*, *MAPK9*, and *IKBKB*) are shared by both diabetes and COVID-19, of which seven genes (*TNF*, *MAPK1*, *MAPK8*, *PIK3CA. PIK3CB*, *PIK3CD*, and *MAPK3*) are the core gene set that is shared among all three groups of datasets (Supplementary Figure S1A). While we analyzed the hubness of these genes using Enrichr PPI Hub Proteins, it was found that *IRS1* is the critical gene (Supplementary Figure S1B and Supplementary Table S1). To understand how these 21 genes are involved in COVID-19 pathogenesis, we considered the top 10 pathways enriched by Enrichr. We mainly considered the microbial infection pathways in this analysis. In Reactome-based Enrichr analysis, the Fc epsilon receptor (FCERI) signaling pathway followed by PI-3K cascade was the top pathway for these shared genes (Supplementary Table S1). However, in KEGG 2019 Human pathway-based analysis, the Fc epsilon RI signaling pathway enriched at rank 12. The insulin signaling pathway, FoxO signaling pathway, HIF-1 signaling pathway, and toll-like receptor (TLR) signaling pathway were the top 10 pathways from the KEGG database (Supplementary Table S1). To find a consensus in pathways, we used the TopFun algorithm of ToppGene suit and found the insulin signaling, toll-like receptor signaling, and Fc epsilon RI signaling pathways commonly enriched within the top 10 pathways (Supplementary Table S1), while we considered the common core seven genes of the three data sets (*TNF*, *MAPK1*, *MAPK8*, *PIK3CA*, *PIK3CB*, *PIK3CD*, and *MAPK3*) and used the same analysis. Similar to the previous analysis, *IRS1* was identified as the key hub gene (Supplementary Figure S1C), and Fc epsilon RI signaling and toll-like receptor signaling pathways were among the top ten pathways (Table 1, Supplementary Table S1).

To find the key targets in the common 21 genes and seven common core genes, we applied the candidate gene prioritization approach using ToppGene. For the 21 genes, 2294 genes from our multi-omics data and KEGG COVID-19 pathway genes were used. The results show that *INSR* and *TNF* are top candidate genes. While we used 225 KEGG COVID-19 pathway genes as the training set, *TNF* is found to be the top candidate gene (Supplementary Table S1). Further, digoxin and ceritinib were the approved drugs found against TNF and INSR, respectively, as per the DTP database [42]. (Supplementary Figure S1D).

Therefore, taken together, we concluded that diabetes or insulin signaling pathway is interacting or sharing the sub-pathways with pro-inflammatory cytokine signaling cascades (TLR, Fc epsilon receptor, FoxO, and HIF-1) associated with COVID-19 pathogenesis. Activation of TLR signaling in SARS-CoV-2 infection leads to the secretion of pro-inflammatory cytokines and cytokine storms [43]. Further, the Fc epsilon RI signaling pathway is also associated with cytokine secretion and inflammatory responses [44]. Therefore, diabetic patients are at risk of developing severe COVID-19 and cytokine storm upon SARS-CoV-2 infection, as reported frequently [45], probably due to activation of these pro-inflammatory cytokine signaling pathways. Further, our analysis with the 21 and seven genes (that link the diabetes pathway with COVID-19) suggests that TNF and INSR are key targets in COVID-19 patients with pre-existing diabetes, and the anti-TNF anti-INSR drugs may be repurposed in managing the COVID-19 severity in diabetic patients (Table 1).

### 3.2. Pathway Crosstalks in Hypertension and COVID-19

We used the KEGG hypertension (renin-angiotensin system) pathway (hsa04614) in this analysis to identify the pathway–pathway interactions between hypertension and COVID-19. Thirteen genes (*NLN*, *CTSA*, *REN*, *AGT*, *AGTR2*, *ANPEP*, *ENPEP*, *MME*, *MRGPRD*, *ACE2*, *ACE*, *AGTR1*, and *MAS1*) were found shared by hypertension and COVID-19. *ACE2*, *ACE*, *AGTR1*, and *MAS1* are the four-core gene set that is shared by all three data sets (Supplementary Figure S2A). *DED1*, *EFT1*, *GNAI2*, and *JAK2* are the hub-genes of both gene sets (Supplementary Figure S2B,C and Supplementary Table S2). While we considered the top 10 pathways common between hypertension and COVID-19 using these common 13 and f4-gene sets, we observed that the renin-angiotensin system, metabolism of angiotensinogen to angiotensin, and peptide hormone metabolism pathways were the top pathways for these shared genes (Supplementary Table S2).

For target identification from the common 13 genes and 4 common core gene sets, 228 KEGG COVID-19 pathway genes and 2075 genes from our multi-omics data (total 2302 genes) were used as the training set. *AGT*, *ANPEP*, *ACE*, *MME*, and *REN* were the top five targets among the 13 common genes, and *ACE* and *AGTR1* are the top two targets among the 4 core gene sets (Supplementary Table S2). Combining the outcomes from both target lists, we selected *ACE*, *AGT*, *AGTR1*, and *REN* as important targets in COVID-19 for hypertension patients. From the DTP database [42], captopril was found to target both ACE and REN (Table 1). No approved drug is retrieved for AGT (Supplementary Figure S2D).

### 3.3. Pathway Crosstalks in Cancers and COVID-19

For cancer, we used the 395 genes available in the KEGG cancer pathway (hsa05200 and subtracted these cancer genes from our multi-omics and KEGG COVID-19 (hsa05171) pathway genes. We found 18 core genes (*STAT1*, *IL6*, *EGFR*, *AGTR1*, *CXCL8*, *NFKB1*, *JUN*, *STAT3*, *MAPK1*, *MAPK8*, *FOS*, *PIK3CA*, *PIK3CB*, *PIK3CD*, *CHUK*, *JAK1*, *NFKBIA*, and *MAPK3*) common to all three datasets (Supplementary Figure S3A). *JAK2*, *STAT3*, *EGFR*, *STAT1*, and *IRS1* are key hub-genes identified from these 18 genes (Supplementary Figure S3B and Supplementary Table S3). Pathway mapping shows that the critical pathways shared between cancer and COVID-19 by these 18 genes were Fc epsilon receptor (FCERI) signaling. Signaling by interleukins, TLR signaling, TRAF6-medi0ated induction of pro-inflammatory cytokines, TNF-α signaling via NF-kB, hepatitis B and C, and AGE-RAGE signaling pathway in diabetic complications. Interleukin, TLR, and TNF signaling are enriched by at least two algorithms (Supplementary Table S3). While we used the 34 genes shared between the KEGG cancer pathway (hsa05200) and the KEGG COVID-19 pathway (hsa05171), we got similar pathways (data not shown).

We used these 18 core common genes and the 16 genes (total 34 genes) shared by the KEGG COVID-19 and cancer pathways for candidate gene prioritization-based target identification. Note that 2208 genes from our multi-omics and KEGG COVID-19 pathway datasets were used in the training gene set. The enrichment shows that *IL6* is the top target, followed by *CXCL8*, *EGFR*, *FOS*, *PIK3R1*, and *NFKB1* (Supplementary Table S3). From the DTP database, prednisolone was found to target IL6, and digoxin was found to target both CXCL8 and NFKB1 (Table 1 and Supplementary Figure S3C).

### 3.4. Pathway Crosstalks in CKDs and COVID-19

Since there is no pathway for CKD in KEGG or Reactome, we used 908 genes associated with CKD from the DisGeNET. We did not consider the gene mutations in this gene list. Following our method, we identified 32 common core genes (*DDX58*, *STAT1*, *IL6*, *NLRP3*, *CYBB*, *EGFR*, *ACE2*, *IL1B*, *F2*, *IFNA1*, *CXCL8*, *CCL2*, *IL2*, *NFKB1*, *MAS1*, *CSF2*, *TLR4*, *JUN*, *STAT3*, *TLR3*, *VWF*, *C3*, *MAPK1*, *ADAM17*, *FOS*, *PIK3CA*, *PIK3CB*, *PIK3CD*, *CASP1*, *MAPK14*, *CSF3*, and *MAPK3*) among the three groups of our data sets (Supplementary Figure S4A). *FOS*, *EGFR*, *STAT3*, *STAT1*, *SYK*, *JAK2*, *AR*, *PTK2*, *PRKCD*, and *JUN* were the top ten hub-genes (Supplementary Figure S4B and Supplementary Table S4).

In pathway mapping of these 32 genes by Enrichr (Reactome, MSigDB, KEGG) and by TopFun, the common pathways ranked within the top 10 were cytokine signaling/inflammatory response, signaling by interleukins/IL-6/JAK/STAT3 signaling, TNF-α signaling via NF-kB, Toll-like receptor signaling pathway, AGE-RAGE signaling pathway, Fc epsilon receptor (FCERI) signaling, etc. (Supplementary Table S4). For target identification from these 32 common core genes, we used 2050 genes from the COVID-19 pathway (KEGG) and our multi-omics datasets. IFNA1 (Interferon-α1) and C3 (Complement 3) were identified as the top two targets (Table 1 and Supplementary Table S4). IFN-α therapy and AMY-101 (C3 blocker) are probable therapeutic options and the drugs associated with other key targets are presented in Supplementary Figure S4C.

### 3.5. Pathway Crosstalks in CVDs and COVID-19

Similar to CKD, no pathway was found in KEGG or Reactome for CVDs. Hence, we gathered 1551 genes from the DisGeNET related to CVDs. In Venny analysis, we identified 39 core common genes (*CXCL10*, *EIF2AK2*, *FGG*, *IL6*, *CFB*, *NLRP3*, *CYBB*, *TLR2*, *EGFR*, *ACE2*, *ACE*, *TNF*, *IL1B*, *F2*, *AGTR1*, *CXCL8*, *CCL2*, *IL6R*, *NFKB1*, *MAS1*, *TLR4*, *JUN*, *STAT3*, *VWF*, *C3*, *MAPK1*, *MAPK8*, *ADAM17*, *FOS*, *MBL2*, *PIK3CA*, *PIK3CB*, *PIK3CD*, *CASP1*, *MAPK14*, *CSF3*, *MAPK3*, *MASP1*, and *TLR8*) shared among the three groups of our data sets (Supplementary Figure S5A). *STAT3*, *EGFR*, *STAT1*, *IRS1*, *JAK2*, *AR*, *RAC1*, *MAPK1*, *HSP90AA1*, and *MAPK3* were identified as the top ten hub-genes (Supplementary Figure S5B and Supplementary Table S5).

Based on the 39 core common genes, immune system, cytokine/inflammatory response, signaling by interleukins, TNF-α signaling via NF-kB, IL-6/JAK/STAT3 signaling, TLR signaling, and AGE-RAGE signaling pathways, etc., are found as links between CVD and COVID-19 (Supplementary Table S5). The top five targets identified based on candidate gene prioritization were *C3*, *CFB*, *EGFR*, *IL6*, and *TLR2* (Table 1 and Supplementary Table S5 and Supplementary Figure S5C), where the 39 common core genes were used as a test set, and 1899 genes from the COVID-19 pathway (KEGG) and our multi-omics datasets were used as training sets. AMY-101 (C3), desipramine (MAPK1), clotrimazole (HSP90AA1), and prednisolone (IL6) were identified as potential drugs (Table 1 and Supplementary Table S5 and Supplementary Figure S5C).

### 3.6. Shared Pathway-Based Individual and Common Targets in COVID-19 and Its Associated Comorbidity

To identify the shared pathway-based individual and common targets, we used InteractiVenn [41] and considered the shared pathway-based top ten targets for each comorbid condition (Supplementary Figure S6). We found that INSR, TNF, INS, PKM, IKBKB, PIK3CD, HK2, and HK3 are key targets in diabetic patients infected with SARS-CoV-2 and digoxin (TNF), ceritinib (INSR), idelalisib (PIK3CD), and felbamate (PKM) may be possible drugs to treat such patients. For COVID-19 patients with existing hypertension, the key targets and drugs are losartan, saralasin, telmisartan for targets AGTR1, AGTR2, and captopril for ACE and REN. For other comorbid conditions, please see Table 2 and Supplementary Figure S7. While we analyzed the targets and drugs for COVID-19 patients having multiple comorbidities, for diabetes, cancer and COVID-19, PIK3R1 was identified as the only target and there is no approved drug identified through DTP [42] search. For diabetes + CVD + CKD + COVID-19, the identified target was MAPK1 and associated drugs are desipramine and guanfacine. For CVD + CKD + COVID-19, the targets were C3, TLR4, MAPK14, and CYBB. Further, sorafenib for MAPK14, and the experimental drug AMY-101 for C3, were identified. In case of CVD + Cancer + CDK + COVID-19, the drugs and targets were lapatinib, gefitinib for EGFR, prednisolone for IL6, and AZD-1480, currently in a phase I/II trial for STAT3. We did not find any common target for complex conditions such as diabetes + hypertension, diabetes + CVD + cancer, diabetes + CKD + cancer, CVD + cancer, CKD + cancer, and diabetes + hypertension + CVD + CKD + cancer (Table 2 and Supplementary Figure S7).

### 3.7. Hub-Gene Based Targets for Specific Comorbidity Associated with COVID-19

In another approach to identify specific targets in individual comorbid conditions, we used the identified top ten hub-genes and InteractiVenn [41]. We separated the individual targets and the common targets for each condition, as shown in Table 3 and Supplementary Figure S8. The IL-6/JAK/STAT3 signaling pathway associated with JAK2 is the common target for all five comorbid conditions. Further, DTP-based retrieval of approved drugs showed that combination therapy with sorafenib, sunitinib, nintedanib (PDGFRB), sunitinib, nintedanib, ruxolitinib (JAK1), and ceritinib (IGF1R) could be helpful to diabetic COVID-19 patients. Similarly, sorafenib (RAF1) and rosiglitazone, tolvaptan (JUN) may be good candidates to manage hypertensive and CKD patients, respectively, infected with SARS-CoV-2. For CVD patients, desipramine (MAPK1) and clotrimazole (HSP90AA1) may be repurposed if infected with SARS-CoV-2. Importantly, nintedanib and ruxolitinib that target JAK2 may be used in COVID-19 cases having any of the five comorbid conditions (Table 3 and Supplementary Figure S8). The targets and corresponding drugs associated with various other combinations of the comorbid conditions are presented in Table 3 and Supplement Table S6 and Supplementary Figure S9.

## 4. Discussion

Previous reports suggest that the TLR pathway, specifically TLR4, is associated with SARS-CoV-2 transmission and COVID-19 severity by activating the hyper-inflammation process [46,47]. It has also been postulated that activation of TLR7 could be effective against COVID-19 [48]. Further, TLR4 signaling is presumed to be associated with COVID-19-related severities in patients with pre-existing comorbidities such as diabetes, obesity, atherosclerosis, hypertension, and aging. TLR pathway is also associated with the pathophysiology of these comorbid conditions [49]. In our analysis, the TLR signaling pathway was found to be shared by COVID-19 and its four comorbid conditions—diabetes, cancers, CKD, and CVDs. Fc epsilon RI signaling was recently reported to be shared by COVID-19 and schizophrenia [50], and the Fc epsilon RI inhibition signaling pathway could be a potential therapeutic option in managing severe COVID-19 [51]. Our analyses show that the Fc epsilon RI signaling pathway was shared by COVID-19 and its associated two comorbid conditions, namely diabetes and CKD. We also found that there was possible crosstalk between COVID-19 and insulin signaling in diabetes. Activation of IL-6/JAK/STAT3 signaling was associated with COVID-19 severity, and IL-6 signaling inhibitors are reported to reduce COVID-19-associated mortality [52,53]. The IL-6/JAK/STAT3 signaling is hyperactivated and therefore is a good target in several cancers [54], chronic kidney diseases [55,56], and cardiovascular diseases [57,58]. Therefore, it is evident that COVID-19 shares the IL-6/JAK/STAT3 axis, and it is associated with several comorbid conditions such as cancers, CVDs, and CKDs, as found in our analyses. Similarly, in our analyses, TNF-α signaling via NF-kB was found to be shared between COVID-19 and its comorbid conditions—cancers, CVDs, and CKDs. TNF-α signaling via NF-kB is a target as it is associated with the inflammation-triggered CVDs [59,60], cancer cell migration and invasion [61,62,63], the pathogenesis of CKDs [64,65,66], and severity of COVID-19 [67,68,69]. In the case of a shared pathway between hypertension and COVID-19, we found that the renin-angiotensin system is the key pathway that links these two diseases. SARS-CoV-2 binds to the human ACE2 receptor through its spike protein to permit the virus to enter the host cell [70,71]. ACE2 is a key enzyme that regulates the renin-angiotensin system associated with hypertension. Therefore, the crosstalk between hypertension and COVID-19 is mediated by the renin-angiotensin system [29,72]. Our analysis also found that the renin-angiotensin system is shared by hypertension and COVID-19. Therefore, pathways deregulated in COVID-19 are also deregulated in its comorbid conditions, and thus, in patients with pre-existing comorbidities, increased severity of COVID-19 is anticipated.

We identified TNF and INSR as key targets for diabetic COVID-19 patients based on our comorbid condition-specific target identification. Further, digoxin that targets TNF and ceritinib that inhibits INSR are found to be potential drugs to manage COVID-19 patients having pre-existing diabetes. Anti-TNF drugs that are given priority for repurposing in COVID-19 management [73,74] and anti-INSR therapy need to be re-evaluated due to conflicting reports [75,76]. We also found that anti-PDGFRB (sorafenib, sunitinib, nintedanib), anti-JAK1 (sunitinib, nintedanib, ruxolitinib), and anti-IGF1R (ceritinib) drugs could be potential therapeutics in SARS-CoV-2-infected diabetic patients based on our hub-gene-based targets and drug identification. The JAK1/JAK2 inhibitor protects autoimmune diabetes in experimental mice [77] and improves the condition of diabetic kidney disease [78]. However, caution of using JAK inhibitors for COVID-19 is necessary [79,80], and additional studies are needed for diabetic COVID-19 patients. Similarly, anti-PDGFRB and anti-IGF1R therapy in COVID-19 patients with or without pre-existing diabetes require further investigations [81].

In our analyses, the renin-angiotensin system, metabolism of angiotensinogen to angiotensin, and peptide hormone metabolism pathways were found to be shared by both hypertension and COVID-19. Pharmacological evidence indicated that defects in the natriuretic peptide hormone system may help develop severe pulmonary disorders in patients suffering from COVID-19 [82]. From these pathways, ACE, AGT, AGTR1, and REN were identified as crucial targets due to other factors overriding this presumed RAS mechanism, targeting natural physiologic control. Antihypertensive drugs may have a protective role [83] and may decrease fatality from COVID-19 in elderly COVID-19 patients [84]. Our identified ACE and REN inhibitor captopril and AGTR1 targeting losartan are potential anti-COVID-19 drugs [85]. Captopril (NCT04355429) and losartan (NCT04312009) are currently under clinical evaluation related to their efficacy in managing COVID-19. In addition to these targets, we identified *GNAI3*, *DED1*, *HSPA1A*, *PCNA*, *RUVBL1*, *EFT1*, *RUVBL2*, and *RAF1* as hub-gene-based targets. Sorafenib has been identified as RAF1 and BRaf protein kinase inhibitor and has been reported to inhibit viral infectivity by downregulating ERK/MAPK signaling pathways during viral infection [86]. Therefore, COVID-19 patients with or without hypertension may be treated with these identified drugs after proper clinical evaluation.

IL6, CXCL8, EGFR, FOS, PIK3R1, and NFKB1 were identified as common targets from the common pathways between cancers and COVID-19 (signaling by interleukins, TLR signaling, TNF-α signaling via NF-kB). Increased expression of IL6 was associated with severity in COVID-19 [87], and corticosteroids such as dexamethasone and prednisone or methylprednisolone downregulated IL6 and showed positive clinical outcomes in patients with severe COVID-19 [88,89,90,91]. The role of NFKB1 in cancer and the inflammatory pathway is well documented [67,91]. It is hypothesized that the NF-κB pathway is a potential target in severe COVID-19 cases, and the inhibition of nuclear translocation of NFKB1 could be a potential therapy [67]. Our analysis of digoxin, a cardiac glycoside, targets both the NFKB1 and chemokine CXCL8. Cardiac glycosides have been reported to inhibit cancer cell proliferation, and digoxin blocks tumor development of various xenotransplanted human cancer cells in mice. Digoxin blocked the transcription factor hypoxia-inducible factor 1 (HIF-1) to inhibit tumor growth [92]. Digitoxin also inhibits cytokine storm triggered by influenza virus infection [93], and it has been reported that digoxin and ouabain inhibit SARS-CoV-2 replication with more than 99% efficacy [93]. Digoxin patients admitted for COVID-19 were closely examined for drug–drug interactions [94]. Therefore, the common targets for cancers and COVID-19 identified in this research may be further explored for their role, targeting drugs, and drug–drug interactions in cancer patients infected with SARS-CoV-2.

Our identified IL-6/JAK/STAT3 signaling is associated with CKDs and severity of COVID-19 [52,53,55,56]. Other identified shared pathways are TNF-α signaling via NF-kB, TLR signaling, AGE-RAGE signaling, and Fc epsilon RI signaling. From these pathways, IFNA1 and C3 were found to be suitable therapeutic candidates, but the role of IFNA1 in CDK is not well understood [95]. However, it has been reported that inborn errors of type I IFN immunity are associated with severe COVID-19 [96] since the low type I IFN activity triggers IL6 and NF-α mediated pro-inflammatory responses and cytokine storm [97]. Recent reports suggest that IFN-α therapy could be a potential management strategy for COVID-19 [98,99]. Therefore, IFN-α treatment for COVID-19 patients with CKDs needs further evaluation. Our second pathway-based identified target, C3, is upregulated in various CKDs [100,101] and COVID-19 [102]. Decreased C3 levels in serum are associated with poor prognosis for COVID-19 patients [103]. Unlike the IL6-mediated cytokine storm, activation of C3 acts as an effector mechanism that aggravates organ injury [104]. The C3-targeting drug candidate (AMY-101) has been successfully used to treat acute respiratory distress syndrome (ARDS)-like complement-mediated inflammatory damage of SARS-CoV-2 infection [104,105]. Therefore, C3 inhibitors may work in COVID-19 patients with pre-existing CKDs. Since IFN-α and C3 are identified as common targets in CKD and COVID-19, these two targets may be specifically explored to treat COVID-19 patients with pre-existing CKDs. In our hub-gene-based analyses, JUN is identified as a potential target, and drugs like rosiglitazone and tolvaptan can target JUN. Similarly, C3 blocker AMY-101 should be tested with caution in managing COVID-19 patients with CDKs.

Like CKDs, in CVDs, C3 was identified as a potential target. Our other identified important pathway-based targets are CFB, IL6, and TLR2. A high level of plasma C3 was associated with increased incidences of CVD in men [106], and increased IL6 signaling was associated with various CVDs [107,108]. On the other hand, decreased C3 is associated with severe COVID-19 [103]. Therefore, like CDK, in CVD, the use of C3-targeting AMY-101 for CVD [104] needs to be validated. Like cancers, IL6 inhibition of corticosteroids such as dexamethasone and prednisone or methylprednisolone [88,89,91] may also be evaluated to manage severe COVID-19 patients suffering from CVDs. In our hub-gene-based analysis, MAPK1 and HSP90AA1 were identified as key targets. Differential expression of HSP90AA1 in a co-expression network reported in severe COVID-19 cases [109] and the MAPK pathway play a crucial role in SARS-CoV-2 infection where p38 MAPK could be a potential target [110]. Therefore, all our identified targets need validation for their role in COVID-19 and CVD interactions and potential as therapeutic targets for drug repurposing.

JAK2 was identified as an important target for all the comorbid conditions we have evaluated. The JAK-STAT is associated with cytokine release syndrome in COVID-19 and targeting this pathway could be a potential therapeutic strategy for COVID-19 [111]. The JAK-STAT pathway was also associated with all our selected COVID-19-associated comorbid conditions. The JAK2/STAT3/SOCS axis plays a crucial role in the development of diabetes, where overexpression and phosphorylation of JAK2 were observed [111]. Increased JAK2 activity was associated with angiotensin II type 1 receptor-mediated hypertension and other cardiovascular diseases [112,113], JAK2 is activated in various myeloproliferative disorders [114,115], and JAK2 is overexpressed in polycystic kidney disease [116]. Therefore, JAK2 could be a potential target [112,113,114,115,116]. Induced expression of JAK2 worsens diabetic kidney disease (DKD) in experimental mice [117], and the JAK1/JAK2 inhibitor baricitinib is highly effective in DKD [118]. The JAK2 inhibitor fedratinib has been reported to reduce mortality from COVID-19 [119] and ruxolitinib (a tyrosine kinase inhibitor) inhibits viral infection by inhibition of JAK1 and JAK2 and other JAK inhibitors can block cytokine release associated with COVID-19-induced cytokine storm [120]. Ruxolitinib is under clinical investigation to evaluate COVID-19-associated lung injury [121], and another JAK2 inhibitor, nintedanib, is being evaluated for SARS-CoV-2-induced pulmonary fibrosis [122]. Taken together, our data suggest that JAK2 inhibitors may be tested in COVID-19 patients with any of the comorbid conditions we investigated here with proper precaution.

## 5. Conclusions

In COVID-19 system biology, there are vital information deficiencies. Ongoing research into multi-omics can provide useful information to address the gaps in respect to personalized COVID-19 management, especially where there is pre-existing comorbidity. We have investigated the potential relationship between COVID-19 and its associated key comorbid conditions and how the comorbid conditions aggravate the severity of COVID-19. Our results suggest that interleukin, TLR, TNF, renin-angiotensin, Fc epsilon RI, insulin signaling, PI3K–Akt signaling pathway, and MAPK signaling are partially shared between COVID-19 and its associated comorbidities. Therefore, this research offers valuable perspectives into COVID-19 pathogenesis and severity in its comorbid conditions. Further, our identified shared pathway-based targets for single or multiple comorbidities associated with COVID-19 can help to develop personalized management strategies. Literature and clinical trial information suggest that the several drugs we have identified are potential therapeutics for COVID-19. However, their efficacy in COVID-19 patients with specific comorbid conditions as reported here needs to be appropriately evaluated for their patient-specific applications.

## Figures and Tables

**Table 1:** Summary of the identified pathway crosstalk between COVID-19 and associated five comorbidities.

Comorbid Conditions	Diabetes	Hypertension	Cancers	Chronic Kidney Diseases (CKDs)	Cardiovascular Diseases (CVDs)
**Pathways shared with COVID-19**	Fc epsilon RI signaling, TLR signaling, Insulin signaling	Renin-angiotensin system, Metabolism of angiotensinogen to angiotensins, Peptide hormone metabolism pathways	Signaling by interleukins, TLR signaling, TNF-α signaling via NF-kB	IL-6/JAK/STAT3 signaling, TNF-α signaling via NF-kB, TLR signaling, AGE-RAGE signaling, Fc epsilon RI signaling	IL-6/JAK/STAT3 signaling, TNF-α signaling via NF-kB, TLR signaling, AGE-RAGE signaling
**Shared pathway-based top ten targets**	INSR, TNF, PIK3R1, INS, PKM, MAPK1, IKBKB, PIK3CD, HK2, HK3	AGT, ANPEP, ACE, MME, REN, CTSA, ENPEP, AGTR1, AGTR2, ACE2	IL6, CXCL8, EGFR, FOS, PIK3R1, NFKB1, STAT3, STAT1, PRKCB, JUN	IFNA1, C3, EGFR, TLR4, TLR3, IL6, MAPK1, MAPK14, CYBB, STAT3	C3, CFB, EGFR, IL6, TLR2, MAPK14, MAPK1, STAT3, CYBB, TLR4
**Shared pathway-based drugs**	Digoxin (TNF); Ceritinib (INSR)	Captopril (ACE, REN); Losartan (AGTR1)	Digoxin (CXCL8, NFKB1); Prednisolone (IL6)	IFN-α therapy (IFNA1); AMY-101 (C3)	AMY-101 (C3); Prednisolone (IL6)
**Hub-gene targets**	PDGFRB, JAK1, IGF1R, SHC1	GNAI3, DED1, HSPA1A, PCNA, RUVBL1, EFT1, RUVBL2, RAF1	−	JUN	MAPK1, HSP90AA1
**Hub-gene targeting drugs**	Sorafenib, Sunitinib, Nintedanib (PDGFRB); Sunitinib, Nintedanib, Ruxolitinib (JAK1); Ceritinib (IGF1R)	Sorafenib (RAF1)	−	Rosiglitazone, Tolvaptan (JUN)	Desipramine (MAPK1); Clotrimazole (HSP90AA1)
**Hub-gene common targets and drugs**	Nintedanib, Ruxolitinib (JAK2)	Nintedanib, Ruxolitinib (JAK2)	Nintedanib, Ruxolitinib (JAK2)	Nintedanib, Ruxolitinib (JAK2)	Nintedanib, Ruxolitinib (JAK2)

The table also provides the shared pathways and those pathway-based targets and drugs for specific comorbidity. Further, the top ten hub-gene-based targets and drugs for individual comorbid conditions are also presented.

**Table 2:** Shared pathway-based targets (from top ten targets) and drugs for individual or combinations of comorbidity in COVID-19. Other drugs for these targets are presented in Figure S7.

Comorbid Condition(s)	Shared Pathway-Based Targets	Approved Drugs
**Diabetes**	INSR, TNF, INS, PKM, IKBKB, PIK3CD, HK2, HK3	Digoxin (TNF); Ceritinib (INSR), Idelalisib (PIK3CD), Felbamate (PKM)
**Hypertension**	AGT, ANPEP, ACE, MME, REN, CTSA, ENPEP, AGTR1, AGTR2, ACE2	Losartan, Saralasin, Telmisartan (AGTR1, AGTR2), Captopril (ACE, REN)
**Cancer**	CXCL8, FOS, NFKB1, STAT1, PRKCB, JUN	Digoxin (CXCLB8, NFKB1), Danthron Rosiglitazone, Tolvaptan (JUN)
**CKD**	IFNA1, TLR3	IFN-α therapy (IFNA1)
**CVD**	CFB, TLR2	−
**Diabetes + Cancer**	PIK3R1	−
**Diabetes + Hypertension**	−	−
**Diabetes + CVD + CKD**	MAPK1	Desipramine, Guanfacine (MAPK1)
**Diabetes + CVD + Cancer**	−	
**Diabetes + CKD + Cancer**	−	−
**CVD + Cancer**	−	−
**CVD + CKD**	C3, TLR4, MAPK14, CYBB	Sorafenib (MAPK14), AMY-101 (C3, experimental);
**CVD + Cancer + CDK**	IL6, EGFR, STAT3	Lapatinib, Gefitinib (EGFR); Prednisolone (IL6), AZD-1480 (STAT3, Phase-I/II)
**CKD + Cancer**	−	−
**Diabetes + Hypertension + CVD + CKD + Cancer**	−	−

**Table 3:** Hub-gene targets and drugs for individual and combinations of comorbidities in COVID-19. Other drugs for these targets are presented in Figure S9.

Comorbid Condition(s)	Hub-Gene Targets	Approved Drugs
**Diabetes**	PDGFRB, JAK1, SHC1, IGF1R	Sorafenib, Sunitinib, Nintedanib (PDGFRB); Sunitinib, Nintedanib, Ruxolitinib (JAK1); Ceritinib (IGF1R)
**Hypertension**	GNAI3, DED1, HSPA1A, PCNA, RUVBL1, EFT1, RUVBL2, RAF1	Sorafenib (RAF1)
**Cancer**	−	−
**CKD**	JUN	Rosiglitazone, Tolvaptan (JUN)
**CVD**	MAPK1, HSP90AA1	Desipramine (MAPK1); Clotrimazole (HSP90AA1)
**Diabetes + Hypertension**	GNAI2	−
**Diabetes + CVD**	RAC1	−
**Diabetes + CVD + Cancer**	IRS1	−
**Diabetes + CKD + Cancer**	PTK2, SYK	Fostamatinib (SYK)
**CVD + Cancer**	MAPK3	Sorafenib (MAPK3)
**CVD + CKD**	AR	Dexamethasone, Prednisolone (AR)
**CVD + Cancer + CDK**	STAT3, STAT1, EGFR	Lapatinib (EGFR); AZD-1480 (Phase-II, STAT3)
**CKD + Cancer**	FOS, PRKCD	Ingenol mebutate (PRKCD)
**Diabetes + Hypertension + CVD + CKD + Cancer**	JAK2	Nintedanib, Ruxolitinib (JAK2)

## Data Availability

Data is available in supplement files.

## Supplementary Materials

The following are available online at https://www.mdpi.com/article/10.3390/biomedicines9050556/s1, Figure S1: Pathway crosstalks between diabetes and COVID-19. (A) The shared genes. (B) The hub-genes among the shared 21 genes. (C) The hub-genes among the core seven shared genes. (D) Identified targets and the FDA-approved drugs against these targets. Figure S2: Pathway crosstalks between hypertension and COVID-19. (A) The shared genes. (B) The hub-genes among the shared 13 genes. (C) The hub-genes among the core seven shared genes. (D) Identified targets and the FDA-approved drugs against key targets. Figure S3: Pathway crosstalks between cancer and COVID-19. (A) The shared genes. (B) The hub-genes among the core 18 shared genes. (C) Identified targets and the FDA-approved drugs against these targets. Figure S4: Pathway crosstalks between CKDs and COVID-19. (A) The shared genes. (B) The hub-genes among the core 32 shared genes. (C) Identified targets and the FDA-approved drugs against these targets. Figure S5: Pathway crosstalks between CVDs and COVID-19. (A) The shared genes. (B) The hub-genes among the core 39 shared genes. (C) Identified targets and the FDA-approved drugs against these targets. Figure S6: Venn diagram showing the individual and common targets from shared pathways. The top 10 targets of each comorbid conditions associated with COVID-19 were used in this analysis. Figure S7: Individual and common targets from shared pathways and approved drugs against these targets. Figure S8: Venn diagram showing the individual and common targets from hub-gene analysis. The top 10 hub genes from each comorbid condition associated with COVID-19 were used in this analysis. Figure S9: Approved drugs associated with the hub-gene targets. Drugs for individual and combinations of comorbidities in COVID-19 are presented. Supplementary Table S1: Shared pathways and targets in diabetes and COVID-19. Supplement Table S2: Shared pathways and targets in hypertension and COVID-19. Supplementary Table S3: Shared pathways and targets in cancer and COVID-19. Supplementary Table S4: Shared pathways and targets in CKD and COVID-19. Supplementary Table S5: Shared pathways and targets in CVD and COVID-19. Supplementary Table S6: Hub-gene-based target analysis.
