# Supplementary material for: Predicting COVID-19—Comorbidity Pathway Crosstalk-Based Targets and Drugs: Towards Personalized COVID-19 Management
Source: Biomedicines. 2021 May 17;9(5):556. doi: 10.3390/biomedicines9050556 (PMC8156524; doi:10.3390/biomedicines9050556)

Supplementary Figures

Fig-S1: Pathway crosstalks between diabetes and COVID-19. (**A**) The shared genes. (**B**) The hub-genes among the shared 21 genes. (**C**) The hub-genes among the core seven shared genes. (**D**) Identified targets and the FDA-approved drugs against these targets.

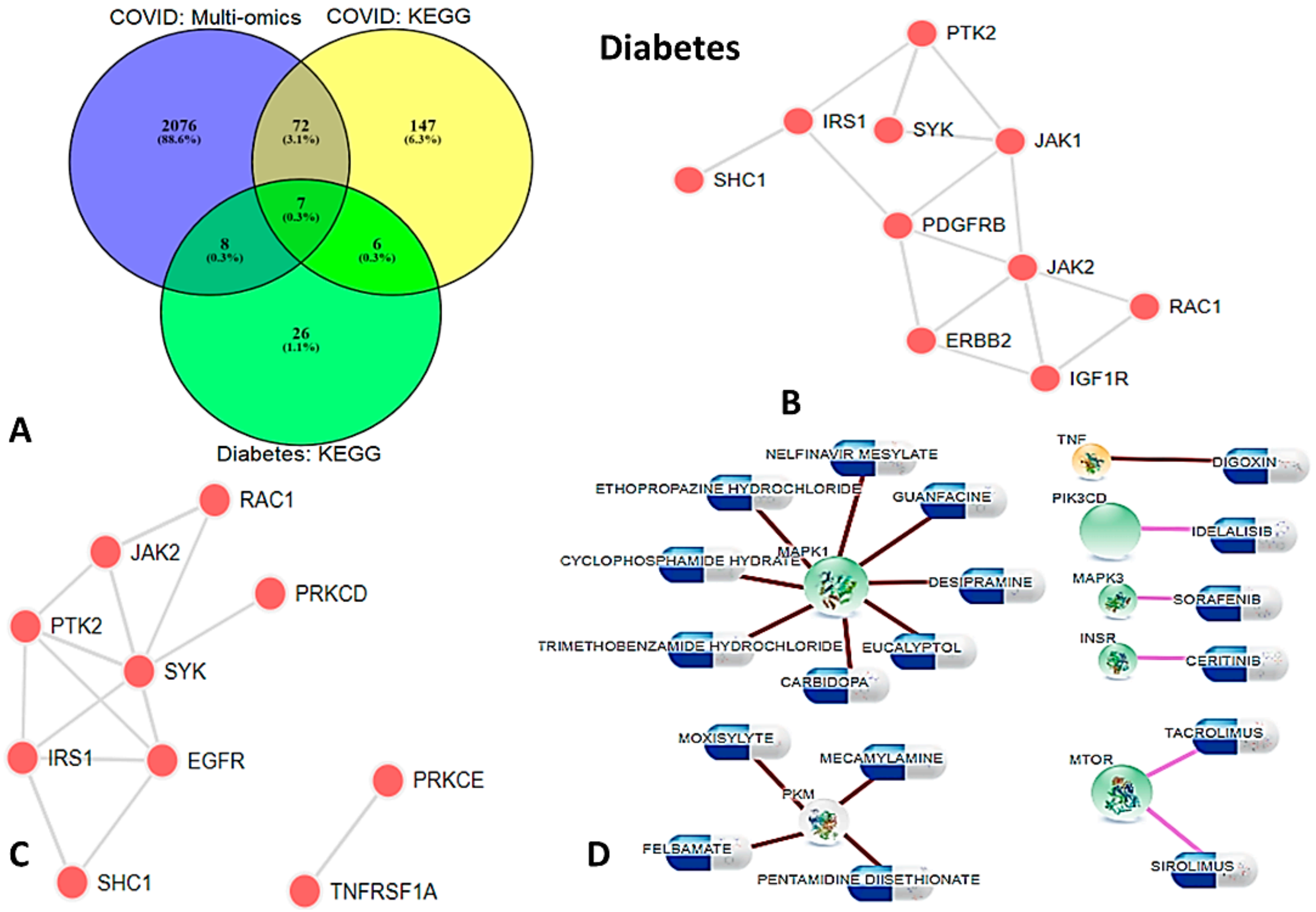

Fig-S2: **Pathway crosstalks between hypertension and COVID-19.** (**A)** The shared genes. (**B)** The hub-genes among the shared 13 genes. (**C)** The hub-genes among the core seven shared genes. (**D)** Identified targets and the FDA-approved drugs against key targets.

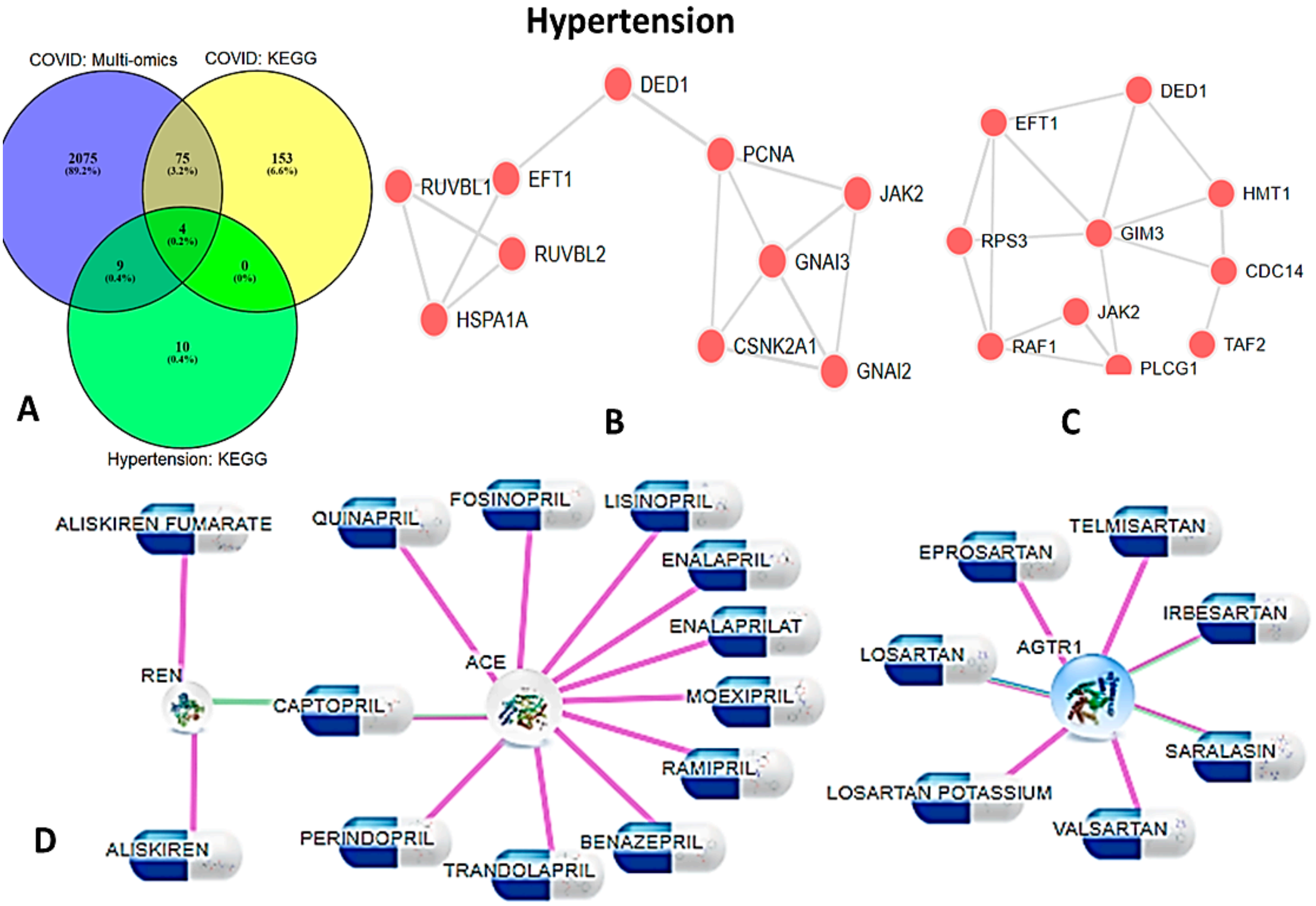

Fig-S3: Pathway crosstalks between cancer and COVID-19. (**A)** The shared genes. (**B)** The hub-genes among the core 18 shared genes. (**C)** Identified targets and the FDA-approved drugs against these targets.

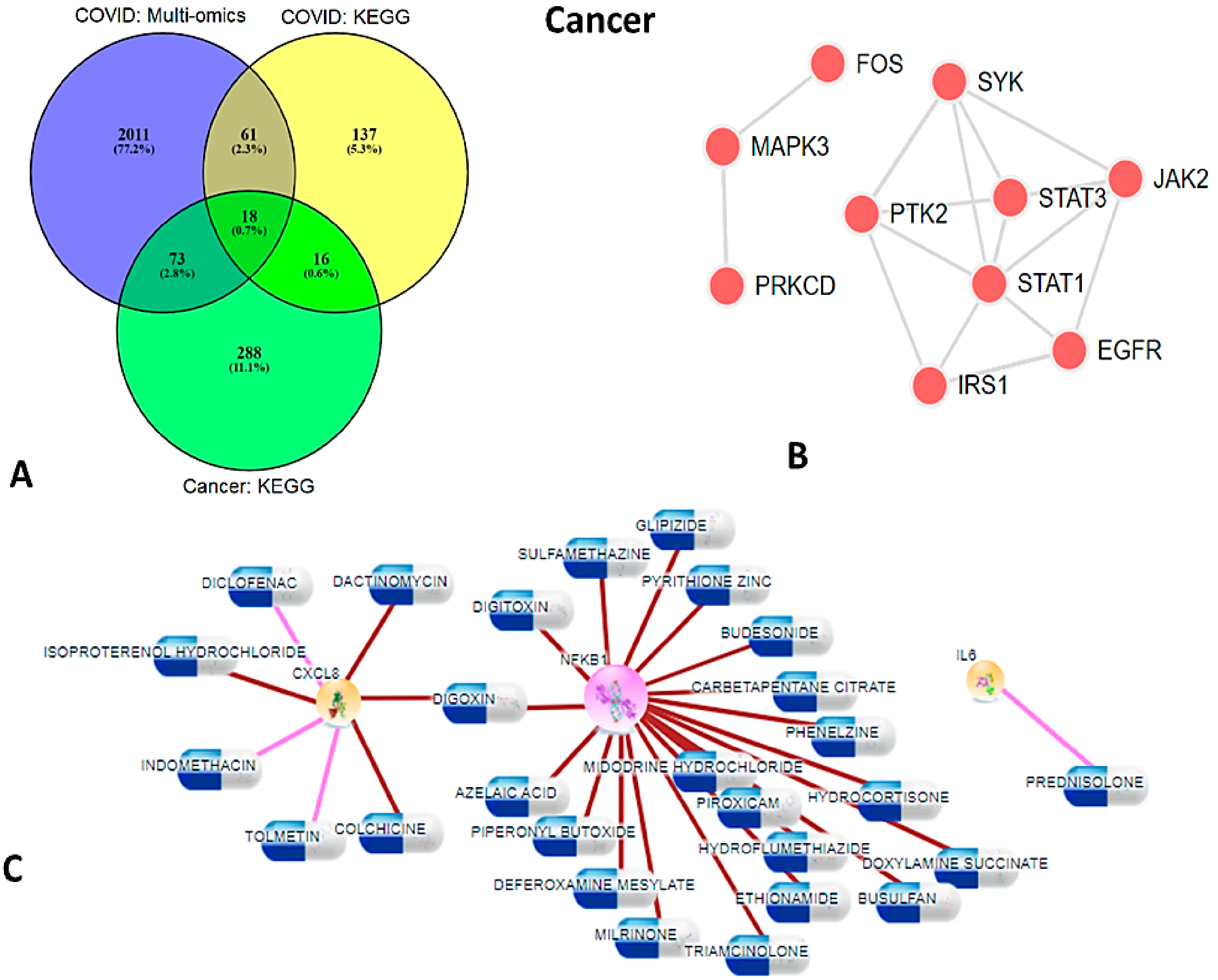

Fig-S4: Pathway crosstalks between CKDs and COVID-19. (A) The shared genes. (**B)** The hub-genes among the core 32 shared genes. (**C)** Identified targets and the FDA-approved drugs against these targets.

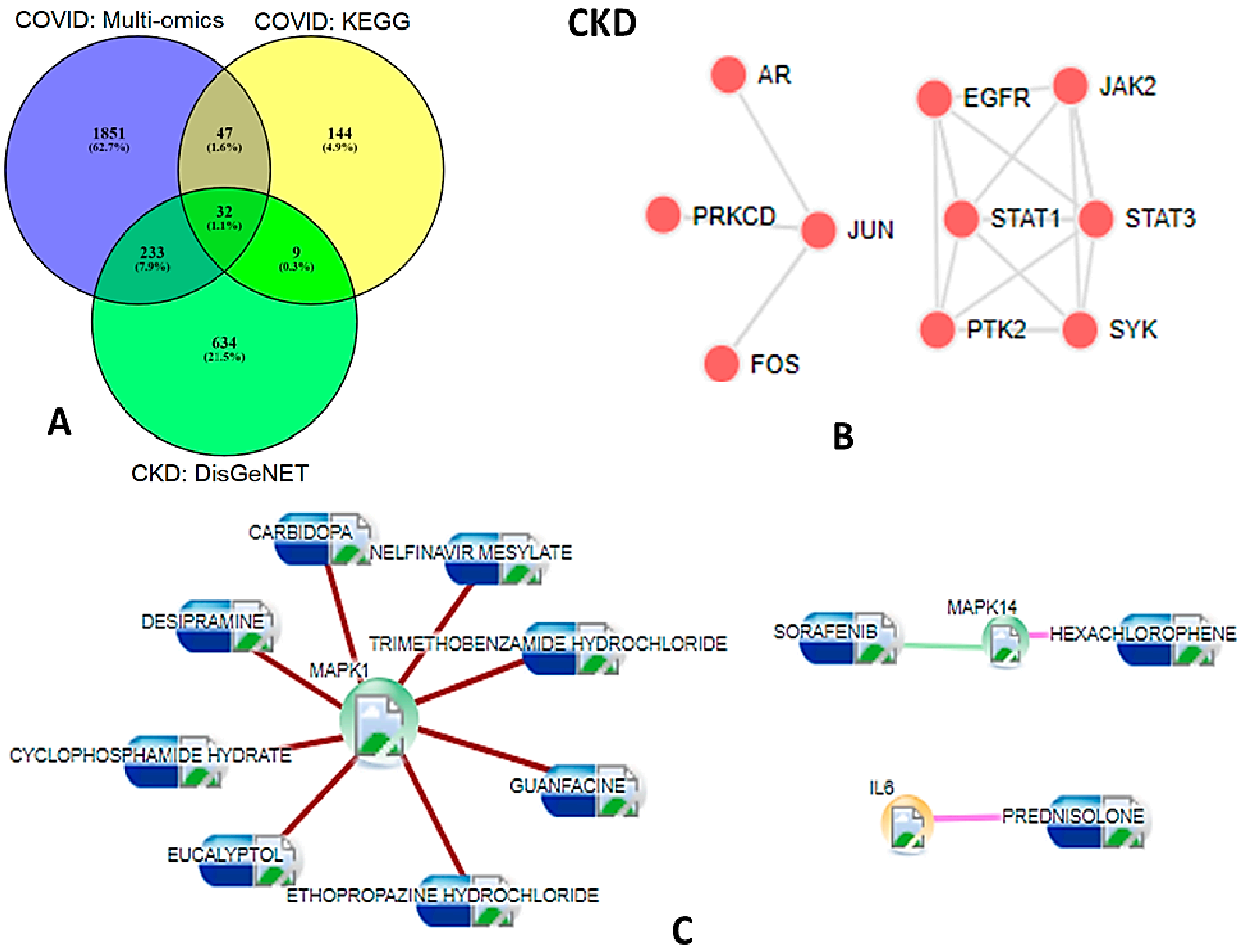

Fig-S5: Pathway crosstalks between CVDs and COVID-19. (A) The shared genes. (**B)** The hub-genes among the core 39 shared genes. (**C)** Identified targets and the FDA-approved drugs against these targets.

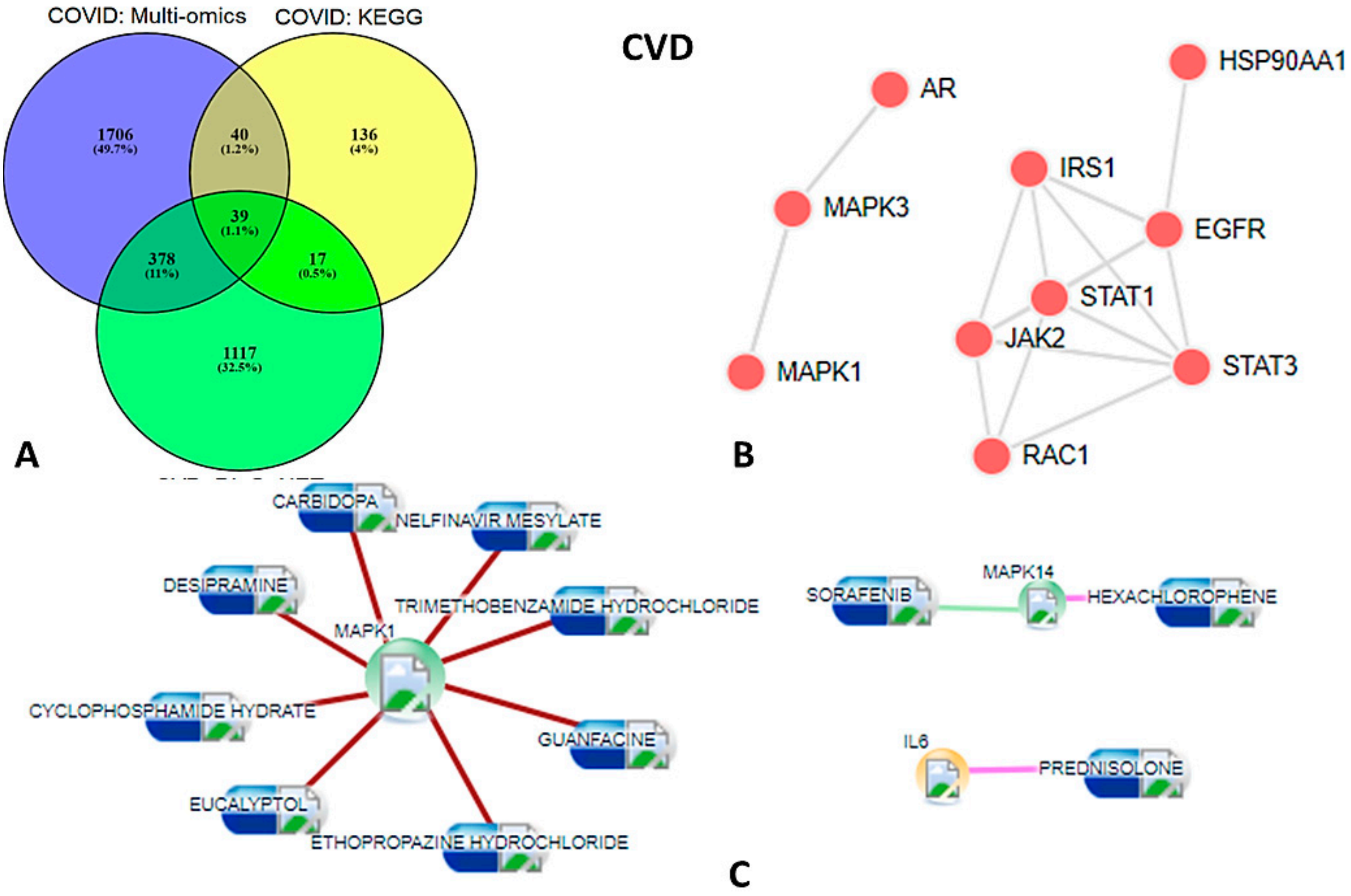

Fig-S6: Venn diagram showing the individual and common targets from shared pathways. Top 10 targets of each comorbid conditions associated with COVID-19 were used in this analysis.

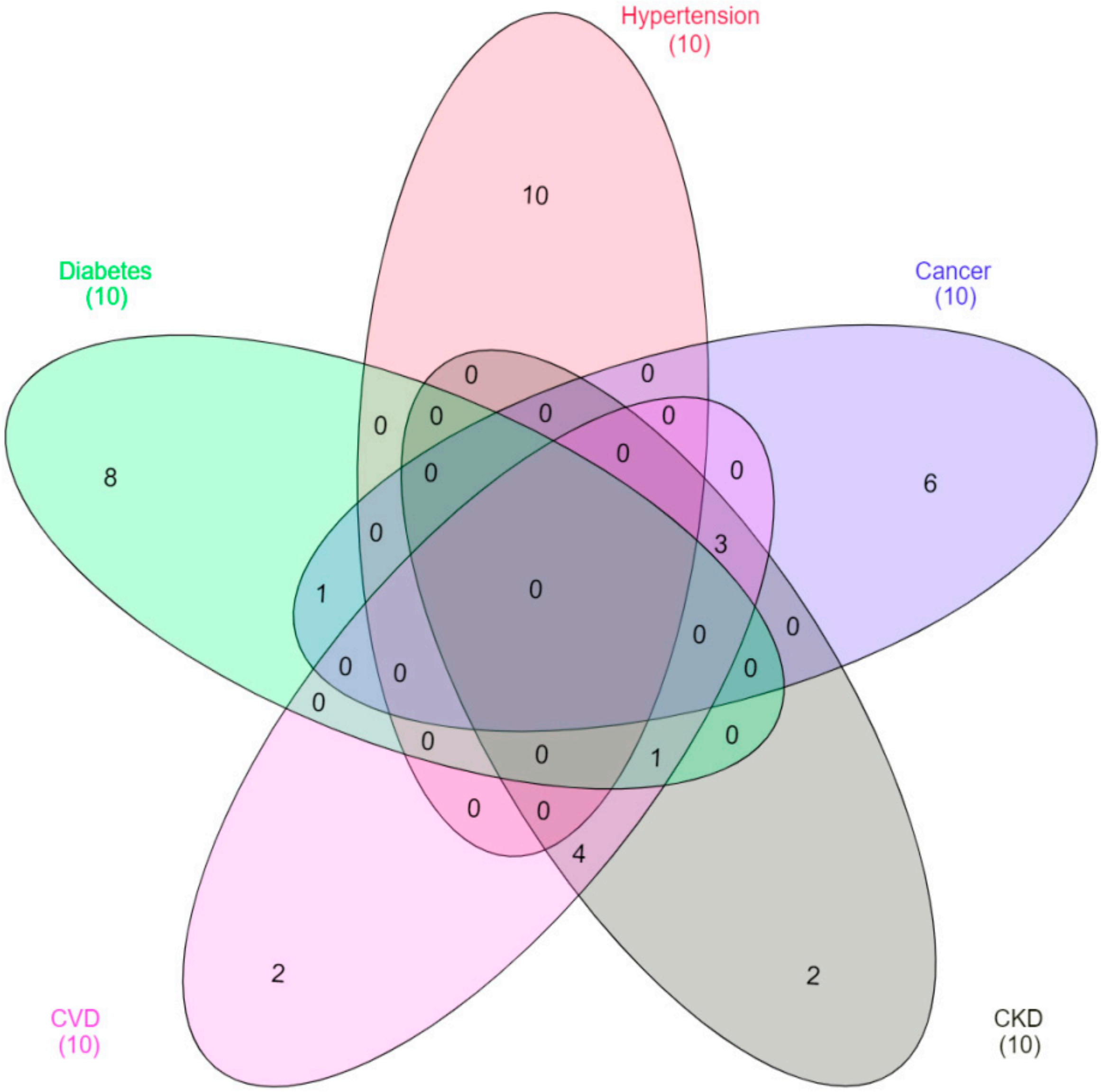

Fig-S7: Individual and common targets from shared pathways and approved drugs against these targets.

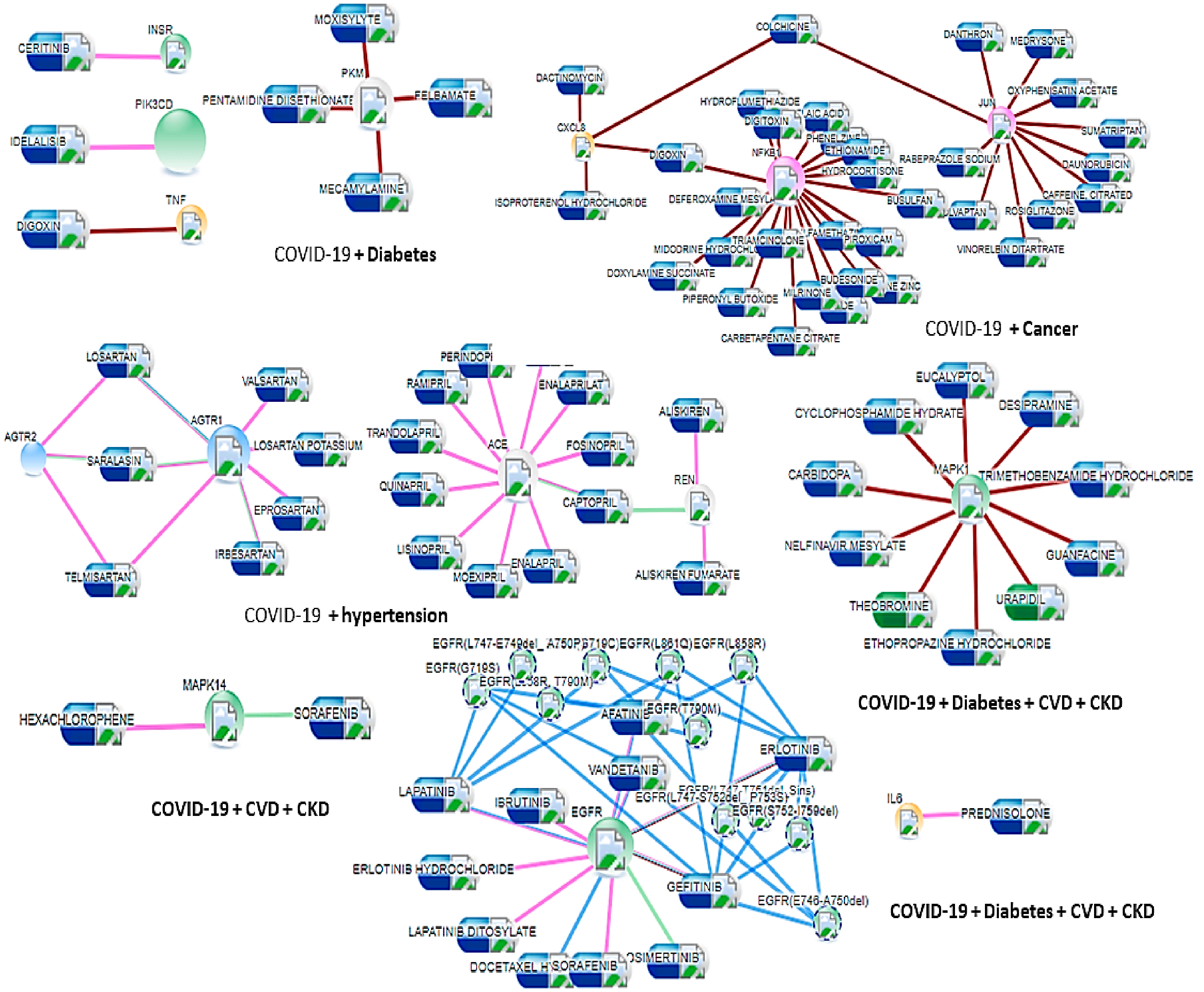

Fig-S8: Venn diagram showing the individual and common targets from hub-gene analysis. Top 10 hub genes from each comorbid condition associated with COVID-19 were used in this analysis.

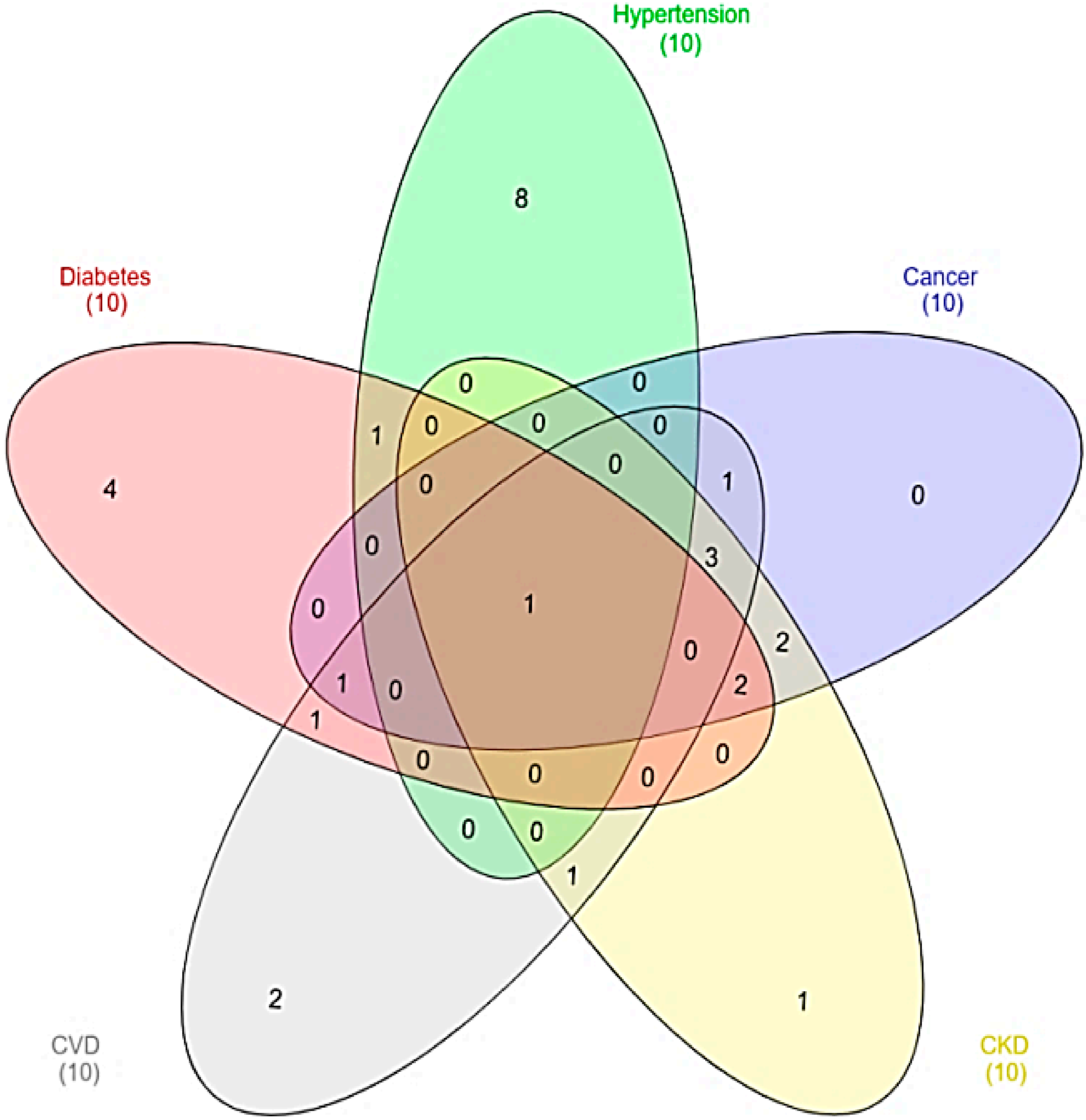

Fig-S9: Approved drugs associated with the hub-gene targets. Drugs for individual and combinations of comorbidities in COVID-19 are presented.

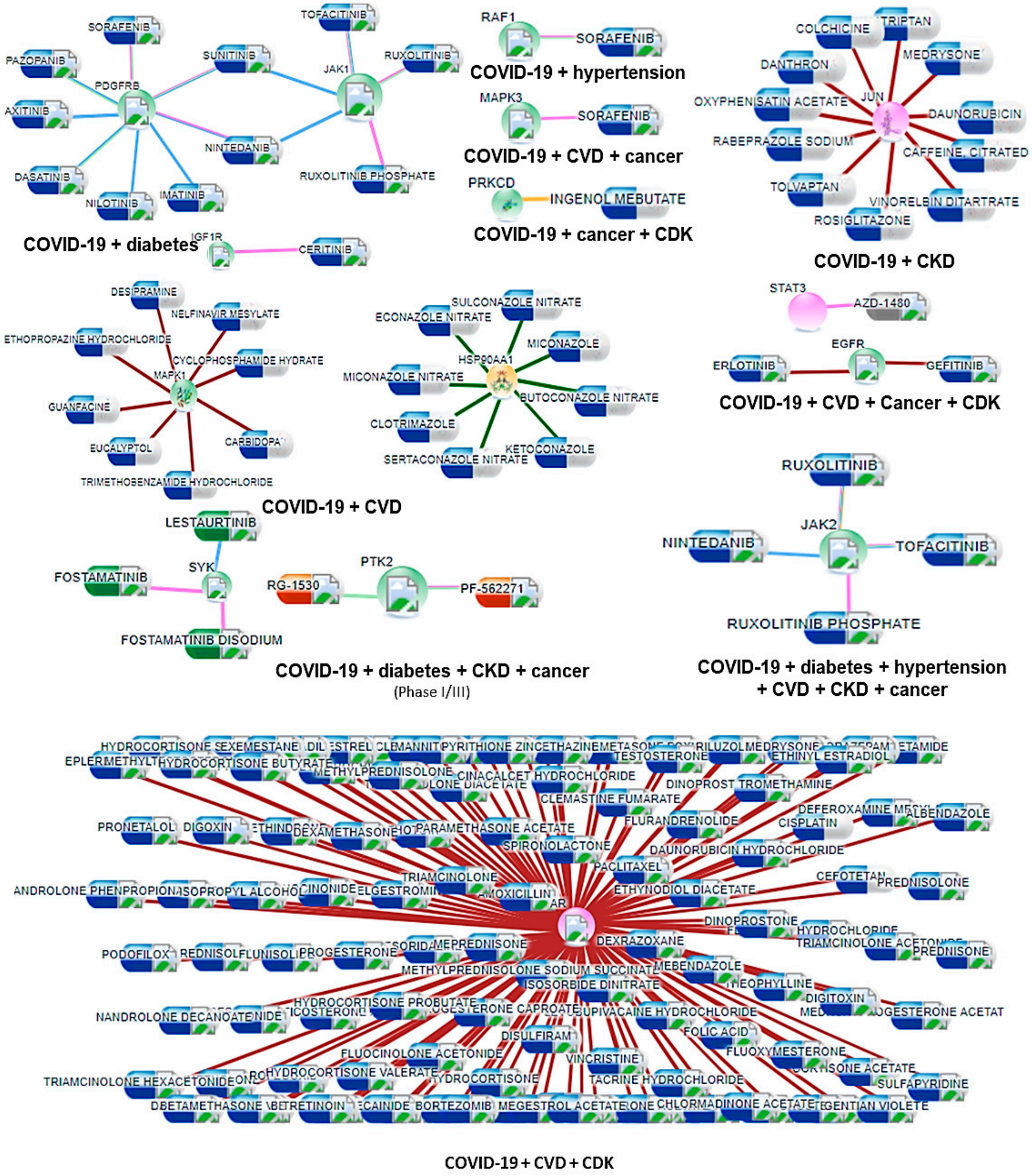

Supplement: Supplementary file 1 [file biomedicines-09-00556-s001.zip › Supplementary Figures.pdf]
